# Psilocybin for treatment-resistant depression: fMRI-measured brain mechanisms

**DOI:** 10.1038/s41598-017-13282-7

**Published:** 2017-10-13

**Authors:** Robin L Carhart-Harris, Leor Roseman, Mark Bolstridge, Lysia Demetriou, J Nienke Pannekoek, Matthew B Wall, Mark Tanner, Mendel Kaelen, John McGonigle, Kevin Murphy, Robert Leech, H Valerie Curran, David J Nutt

**Affiliations:** 1 Psychedelic Research Group, Psychopharmacology Unit, Centre for Psychiatry, Department of Medicine, Imperial College London, W12 0NN London, UK; 2 Computational, Cognitive and Clinical Neuroscience Laboratory (C3NL), Department of Medicine, Imperial College London, W12 0NN London, UK; 3 Cardiff University Brain Research Imaging Centre (CUBRIC), School of Physics and Astronomy, CF24 4HQ Cardiff, UK; 4 Clinical Psychopharmacology Unit, University College London, WC1E 6BT, London, United Kingdom; 5 Imanova Centre for Imaging Sciences, Burlington Danes Building, Hammersmith Hospital, Du Cane Road, London, W12 0NN UK; 6 Investigative Medicine, Department of Medicine, Imperial College London, London, United Kingdom; 7 SU/UCT MRC Unit on Risk and Resilience in Mental Disorders, Department of Psychiatry and Mental Health, University of Cape Town, South Africa

## Abstract

Psilocybin with psychological support is showing promise as a treatment model in psychiatry but its therapeutic mechanisms are poorly understood. Here, cerebral blood flow (CBF) and blood oxygen-level dependent (BOLD) resting-state functional connectivity (RSFC) were measured with functional magnetic resonance imaging (fMRI) before and after treatment with psilocybin (serotonin agonist) for treatment-resistant depression (TRD). Quality pre and post treatment fMRI data were collected from 16 of 19 patients. Decreased depressive symptoms were observed in all 19 patients at 1-week post-treatment and 47% met criteria for response at 5 weeks. Whole-brain analyses revealed post-treatment decreases in CBF in the temporal cortex, including the amygdala. Decreased amygdala CBF correlated with reduced depressive symptoms. Focusing on a priori selected circuitry for RSFC analyses, increased RSFC was observed *within* the default-mode network (DMN) post-treatment. Increased ventromedial prefrontal cortex-bilateral inferior lateral parietal cortex RSFC was predictive of treatment response at 5-weeks, as was decreased parahippocampal-prefrontal cortex RSFC. These data fill an important knowledge gap regarding the post-treatment brain effects of psilocybin, and are the first in depressed patients. The post-treatment brain changes are different to previously observed acute effects of psilocybin and other ‘psychedelics’ yet were related to clinical outcomes. A ‘reset’ therapeutic mechanism is proposed.

## Introduction

Psilocybin is the prodrug of psilocin (4-OH-dimethyltryptamine), a non-selective serotonin 2A receptor (5-HT2AR) agonist and classic ‘psychedelic’ drug^1^. Both compounds occur naturally in the ‘psilocybe’ genus of mushrooms, and are structurally related to the endogenous neurotransmitter serotonin (5-OH-tryptamine, 5-HT). Psilocybin has an ancient and more recent history of medicinal-use. Administered in a supportive environment, with preparatory and integrative psychological care, it is used to facilitate emotional breakthrough and renewed perspective^2^. Accumulating evidence suggests that psilocybin with accompanying psychological support can be used safely to treat a range of psychiatric conditions^1^, including: end-of-life anxiety and depression^3–5^, alcohol and tobacco addiction^6,7^, obsessive compulsive disorder^8^, and most recently from our group, treatment-resistant major depression^9^. Findings from healthy volunteer studies^10^ and trials with other psychedelics^11–13^ supplement those from clinical studies showing that these drugs can have a rapid and lasting positive impact on mental health, often after just one or two doses. Such outcomes raise a number of important questions, including: what brain mechanisms mediate these effects?

Most human functional neuroimaging studies of psychedelics have focused on their acute effects with the aim of elucidating the neural correlates of the ‘psychedelic state’^14,15^. Consistent with findings from animal research^16^, psychedelics appear to dysregulate cortical activity^14,17^, producing an ‘entropic’ brain state^18^, characterised by compromised modular but enhanced global connectivity - referred to previously as network ‘disintegration’ and ‘desegregation’^14^. These effects have been found to correlate with important aspects of the ‘psychedelic experience’, including ‘ego-dissolution’^14,17,19^, and were predictive of post-acute changes in the personality domain ‘openness’^20^. To our knowledge, only one other very recent study has investigated >12 hour post-acute effects of psychedelics on human brain function (although see^12^ and now^21^), and few have looked at anatomical changes possibly related to psychedelic use^22,23^.

The present study focused on changes in brain function before versus after psilocybin in patients with treatment-resistant depression who received two doses of the drug (10 mg followed by 25 mg, one-week apart) as part of an open-label clinical trial. Arterial spin labelling (ASL) and blood oxygen level dependent (BOLD) resting state functional connectivity (RSFC), were used to measure changes in cerebral blood flow (CBF) and functional connectivity before (baseline) and one-day after treatment with psilocybin (i.e. one day after the 25 mg dose). It has been suggested that the days subsequent to a psychedelic experience constitute a distinct phase, referred to as the ‘after-glow’, that is characterised by mood improvements and stress relief^24^. The rationale for scanning one-day post-treatment was to capture brain changes related to this so-called after-glow that might correlate with current mood improvements and/or longer-term prognoses. We predicted that resting-state CBF and FC would be altered post treatment and correlate with immediate and longer-term clinical improvements.

With regards to ‘longer-term’ clinical outcomes, we chose to focus on a 5-week post-treatment endpoint due to a virtual 50:50 split between responders and non-responders at this time-point (QIDS-SR16) and that none of the patients went on to additional (and thus, confounding) treatments within this time frame. A select number of regions of interest were chosen a priori for CBF and RSFC analyses due to previous work implicating their involvement in depression and its treatment, e.g^25–27^.

## Results

Nineteen patients with diagnoses of treatment resistant major depression completed pre-treatment and one-day post-treatment fMRI scanning. Excessive movement or other artefact meant that three patients were removed from the ASL analyses and four from the RSFC (SI Appendix), leaving sample sizes of 16 (mean age = 42.8 ± 10.1 y, 4 females) and 15 (mean age = 42.8 ± 10.5 y, 4 females) for the ASL and BOLD analyses, respectively.

Treatment with psilocybin produced rapid and sustained antidepressant effects. For the patients included in the ASL analysis (minus one patient whose scan 1 rating was not collected), the mean depression score (QIDS-SR16) for the week prior to the pre-treatment scan was 16.9 ± 5.1, and for the day of the post-treatment scan, it was 8.8 ± 6.2 (change = −8.1 ± 6, t = −5.2, p < 0.001). The mean QIDS-SR16 score at baseline (screening) was 18.9 ± 3, and for 5-weeks post-treatment, it was 10.9 ± 4.8 (change = −8 ± 5.1, t = −6.3, p < 0.001). Mean change values for those included in the BOLD analyses were −7.3 ± 5.3 (change from scan 1 to scan 2) and −8.2 ± 5.2 (change from baseline to 5 weeks post-treatment). Both contrasts were highly significant (t = −5.2 and −6.2, p < 0.001). Six of the 15 (BOLD) and 16 (ASL) patients met criteria for treatment response (≤50% reductions in QIDS-SR16 score) at 5 weeks. Of the full 19 patients, *all* showed some decrease in depressive symptoms at 1 week, with 12 meeting criteria for response (change = −10.2 ± 5.3, t = −6.4, p < 0.001). All but one patient showed some decrease in QIDS-SR16 score at week 5 (with one showing no change) and 47% met criteria for response (change = −9.2 ± 5.6, t = −6.7, p < 0.001).

**Figure 1:**
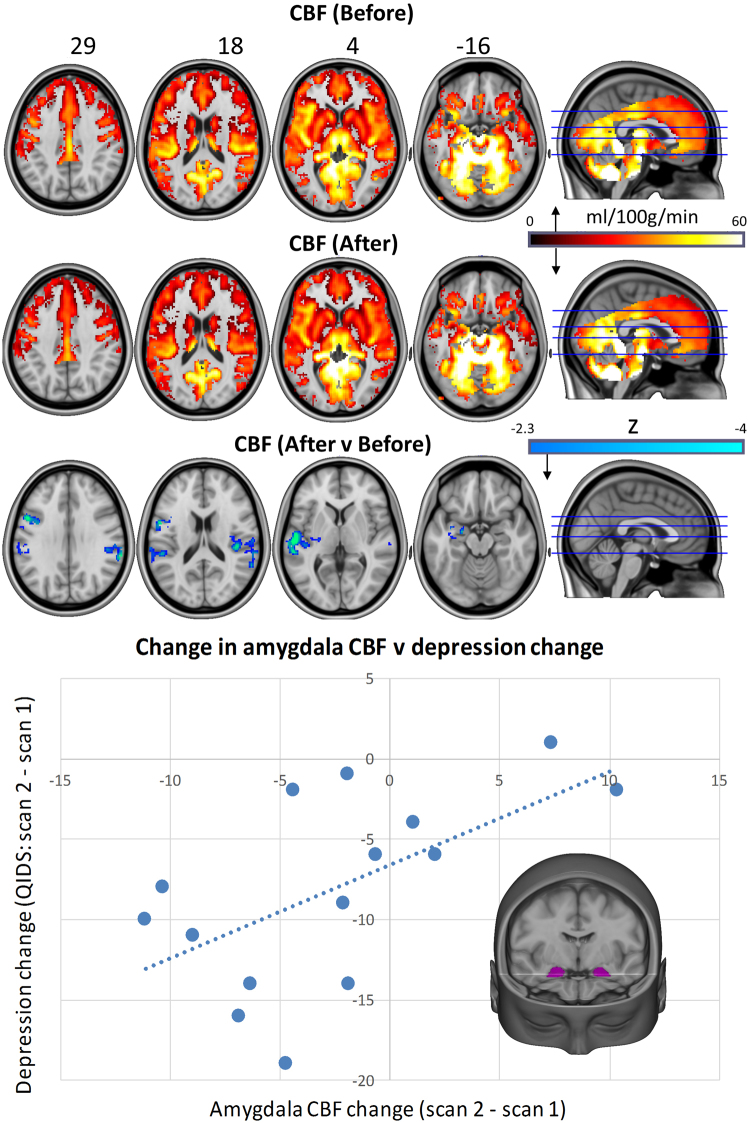
Whole-brain cerebral blood flow maps for baseline versus one-day post-treatment, plus the difference map (cluster-corrected, p < 0.05, n = 16). Correlation chart shows post-Treatment changes in bilateral amygdala CBF versus changes in depressive symptoms (r = 0.59, p = 0.01). One patient failed to completed the scan 2 QIDS-SR16 rating, reducing the sample size to n = 15 for the correlation analysis. In all of the images, the left of the brain is shown on the left.

Whole-brain CBF was calculated pre and post treatment and contrasted (Fig. 1). Only decreases in CBF were observed post treatment (vs pre), and these reached statistical significance in the left Heschl’s gyrus, left precentral gyrus, left planum temporale, left superior temporal gyrus, left amygdala, right supramarginal gyrus and right parietal operculum (Table S1). Based on previous findings of increased amygdala blood flow and metabolism in depression^25^, reductions in amygdala CBF were compared with the reductions in depressive symptoms between scan 1 and 2 (i.e. decreased depressed mood at the time of scanning), and a significant relationship was found (r = 0.59; p = 0.01). After splitting the sample into responders and non-responders at 5-weeks post-treatment, and then comparing CBF changes in a t-test, no significant difference was found (t = 0.11; p = 0.46).

**Figure 2:**
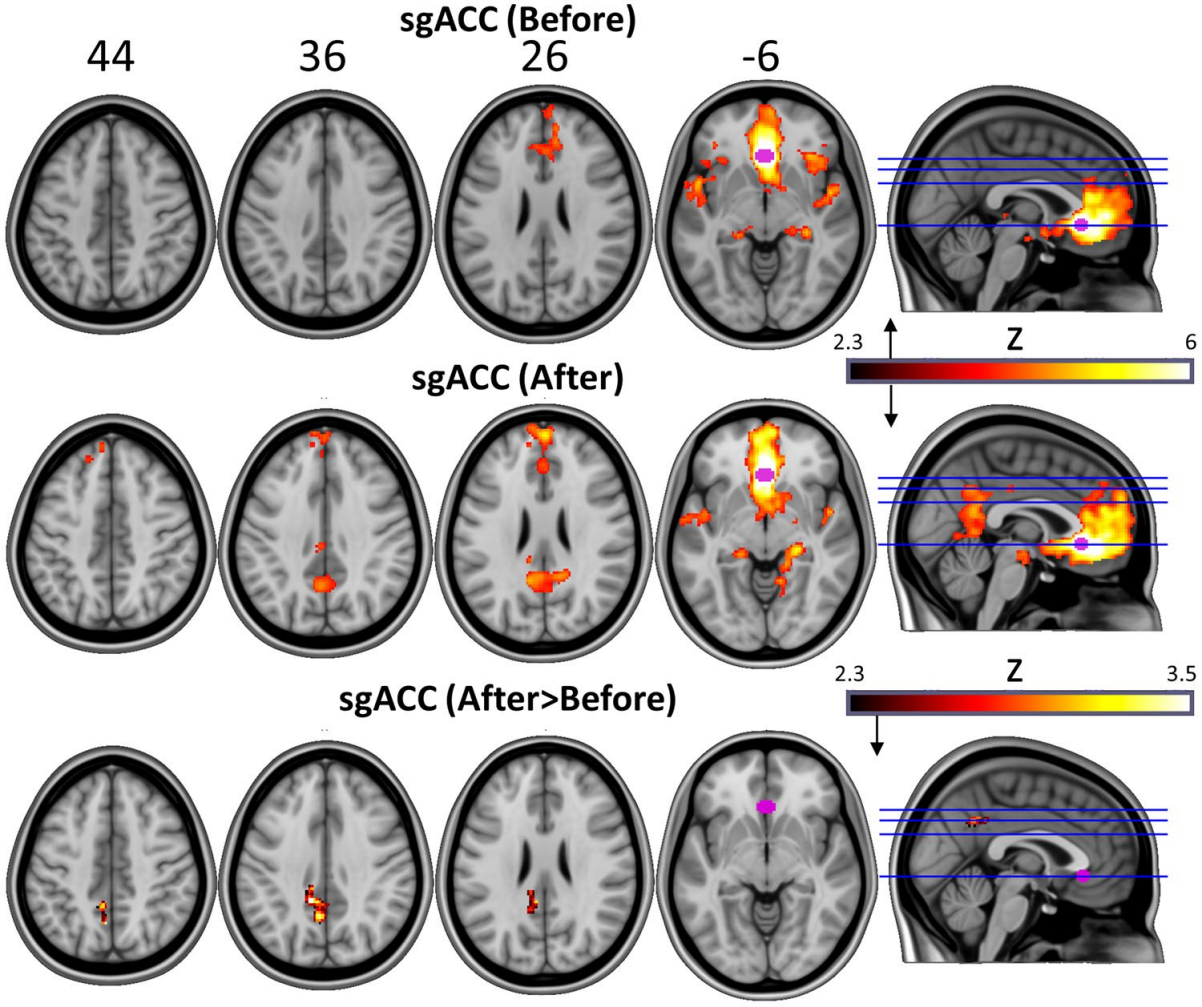
Top two rows = sgACC (purple) RSFC before and after psilocybin treatment (hot colours = regions of significantly positive coupling). Bottom row reveals regions where there was a significant increase in sgACC RSFC post-treatment (hot colours). All maps are cluster-corrected, p < 0.05, Z > 2.3.

**Figure 3:**
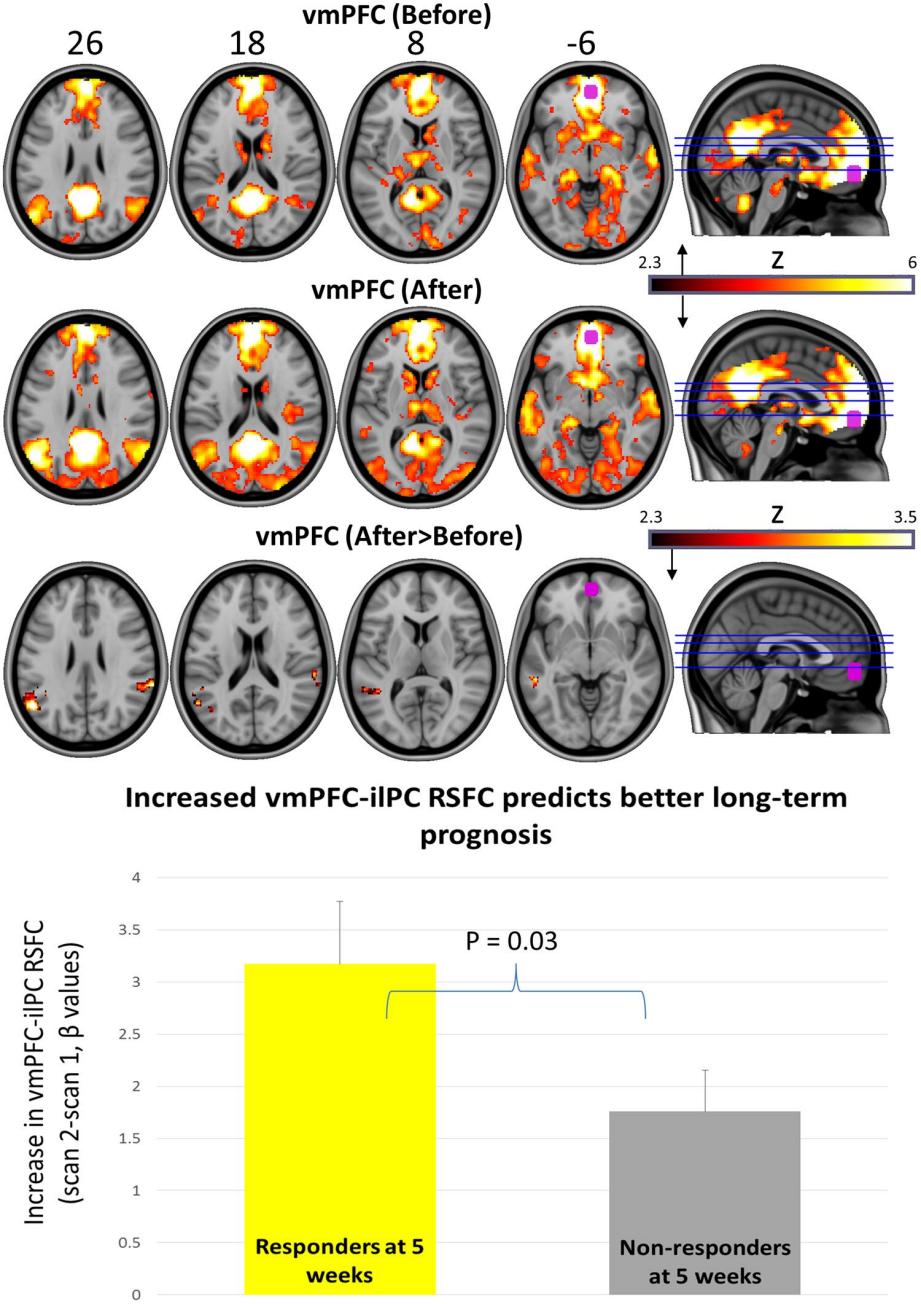
Top two rows = vmPFC (purple) RSFC before and after psilocybin treatment (hot colours = regions of significantly positive coupling). Bottom row reveals regions where there was a significant increase in vmPFC RSFC post-treatment (hot colours). All maps are cluster-corrected, p < 0.05, Z > 2.3. Increased coupling between the vmPFC and the displayed regions (bottom row) was predictive of clinical response at 5-weeks post-treatment. Chart shows mean values and positive standard errors.

**Figure 4:**
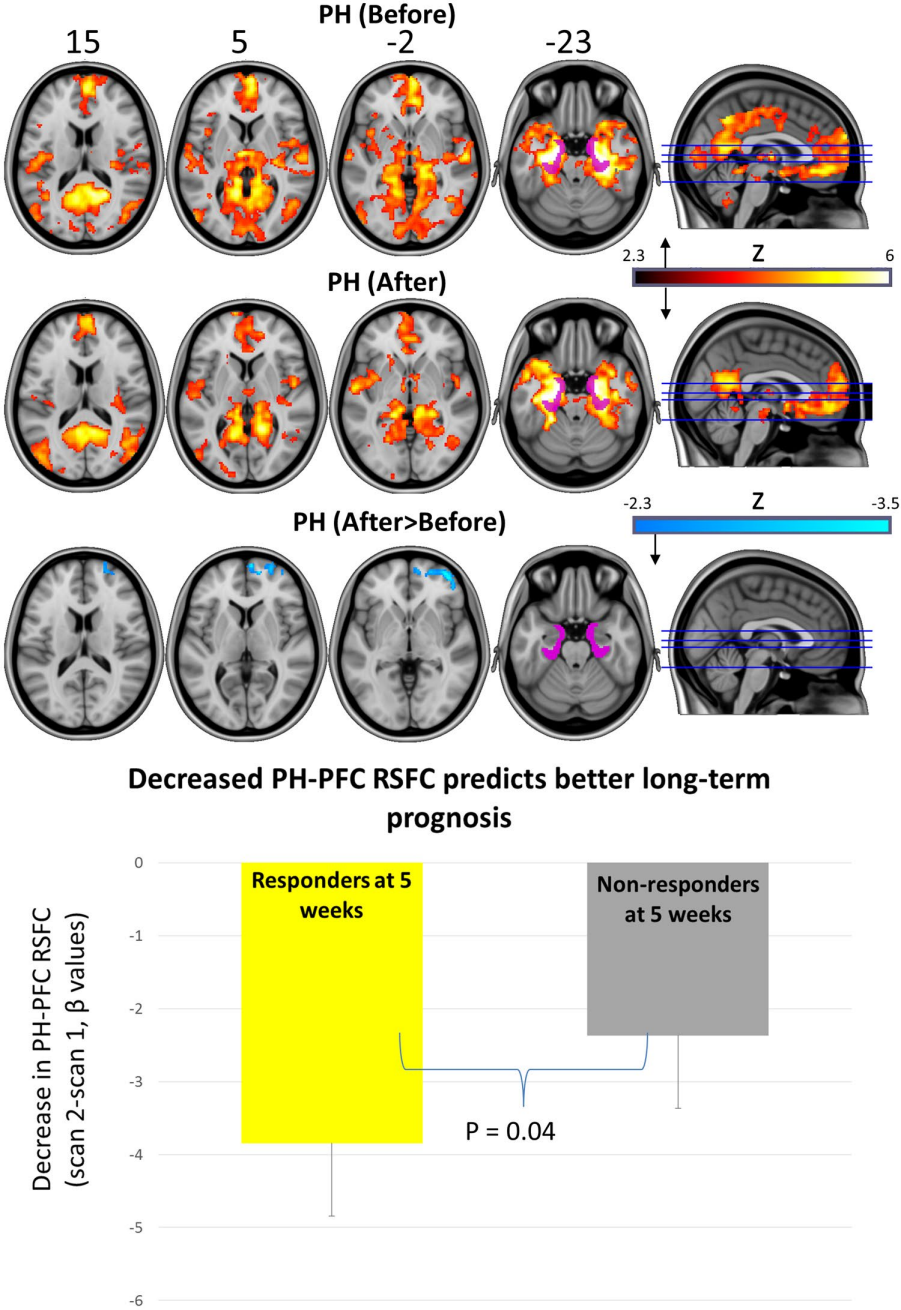
Top two rows = Bilateral PH (purple) RSFC before and after psilocybin treatment (hot colours = regions of significantly positive coupling). Bottom row reveals regions where there was a significant decrease in PH RSFC post-treatment (cold colours). All maps are cluster-corrected, p < 0.05, Z > 2.3. Decreased coupling between the PH and the displayed regions (bottom row) was predictive of clinical response at 5-weeks post-treatment (t = −1.9, p = 0.04). Chart shows mean values and negative standard errors.

Next, seed-based RSFC analyses were performed using the BOLD data. Based on previous data implicating their involvement in the pathophysiology of depression and response to treatments^25–27^, four regions of interest (ROIs) were chosen: 1) the subgenual anterior cingulate cortex (sgACC), 2) the ventromedial prefrontal cortex (vmPFC), 3) the bilateral amygdala, and 4) the bilateral parahippocampus (PH) (Figs 2–4 and SI Appendix, Table S1).

Increased sgACC RSFC was observed with the posterior cingulate cortex/precuneous (PCC) post-treatment (Fig. 2) but this effect did not correlate with reductions in depressive symptoms between scan 1 and 2 (r = −0.2; p = 0.24) and nor did it predict treatment response at 5 weeks (t = −1.3; p = 0.11).

Increased vmPFC RSFC was observed with the bilateral inferior-lateral parietal cortex (ilPC) post-treatment. This effect did not correlate with reductions in depressive symptoms between scan 1 and 2 (r = −0.26; p = 0.17) but did predict treatment response at 5 weeks, with responders showing significantly greater vmPFC-ilPC RSFC increases than non-responders (t = 2.1; p = 0.03).

Decreased PH RSFC was observed with a PFC cluster incorporating the lateral and medial prefrontal cortex. This effect did not correlate with reductions in depressive symptoms between scan 1 and 2 (r = 0.08; p = 0.38) but did relate to treatment response at 5 weeks, with responders showing significantly greater PH-PFC RSFC decreases than non-responders (t = −1.9, p = 0.04). Amygdala RSFC was not significantly altered post treatment.

**Figure 5:**
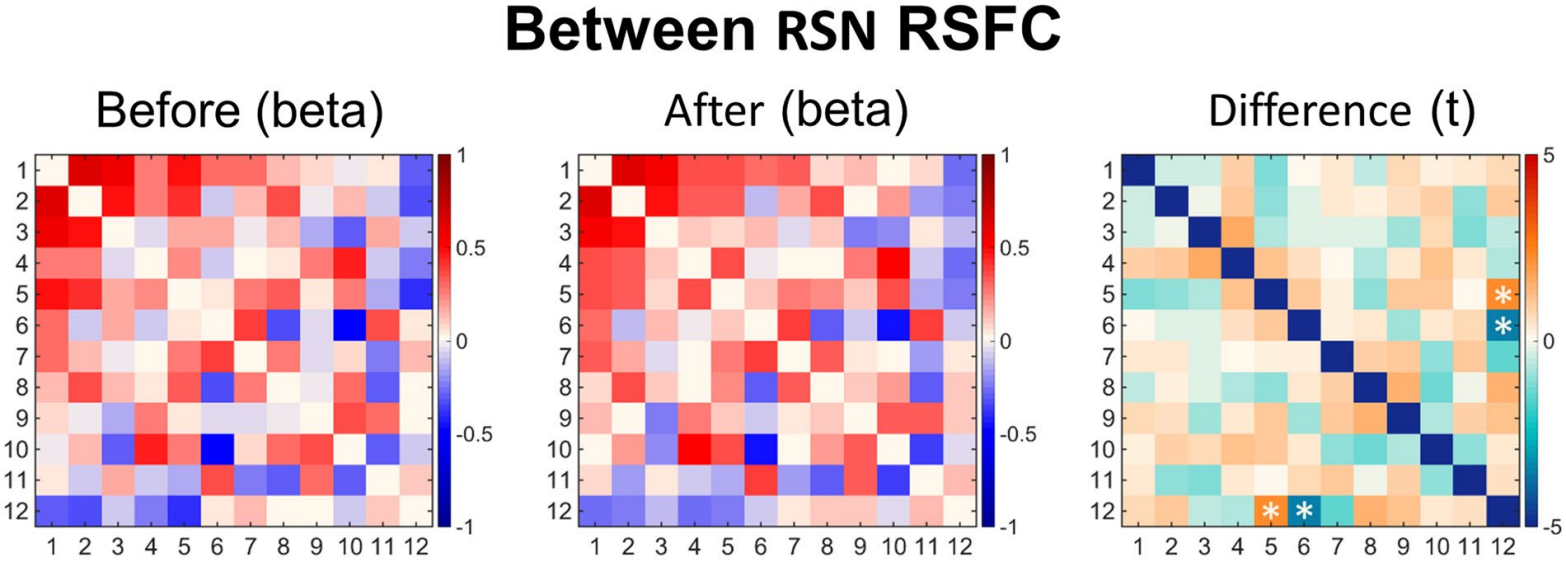
Differences in between-RSN RSFC or RSN ‘segregation’ before and after therapy. Each square in the matrix represents the strength of functional connectivity (positive = red, negative = blue) between a pair of different RSNs (beta values). The matrix on the far right displays the between-condition differences in covariance (t values). The RSNs are: 1) medial visual network, 2) lateral visual network, 3) occipital pole network, 4) auditory network, 5) sensorimotor network, 6) DMN, 7) parietal cortex network, 8) the dorsal attention network, 9) the salience network, 10) posterior opercular network, 11) left frontoparietal network, 12) right frontoparietal network. White asterisks represent significant differences (P < 0.05, non-corrected). Both of the significant differences did not survive FDR correction for multiple comparisons.

Analyses of within network RSFC using 12 previously identified canonical RSNs^14^ revealed increased default-mode network (DMN) (t = 2.7, p = 0.018), dorsal attention network (DAN) (t = 2.2, p = 0.042), and posterior opercular network (POP) (t = 2.7, p = 0.016) RSFC post-treatment; however, these changes failed to survive Bonferonni correction for multiple comparisons (revised α = 0.05/11 = 0.0042) and did not correlate with depression outcomes, e.g. the relationship between change in DMN RSFC and reduced QIDS-SR16 scores between scan 1 and 2 were non-significant (r = 0.25; p = 0.18) and neither were changes in DMN RSFC predictive of outcomes at 5 weeks (t = 0.58; p = 0.28). Analyses of between network RSFC using the same 12 RSNs, revealed decreased RSFC between the DMN and right frontoparietal network (rFP) (t = −3.6, p = 0.0031) and increased RSFC between the sensorimotor network (SM) and rFP (t = 2.2, p = 0.045) (Fig. 5); however, these effects did not survive FDR correction for multiple comparisons and did not relate to reduced QID-16 scores between scan 1 and 2, nor response at 5 weeks.

Lastly, based on indications from previous work^4,5,10^ we explored the possibility that the quality of the acute ‘psychedelic’ experience may have mediated the post-acute brain changes. We focused on a rating scale factor related to ‘peak’ or ‘mystical’ experience and used scores for the high-dose psilocybin session as a covariate in a PH RSFC analysis. The PH was specifically chosen due to previous work implicating its involvement in related states^14^. Results revealed that patients scoring highest on ‘peak’ or ‘mystical’ experience had the greatest decreases in PH RSFC in limbic (e.g. bilateral amygdala) and DMN-related cortical regions (e.g. the PCC). See the supplementary file for the relevant maps and discussion.

## Discussion

The present study goes some way to addressing an important knowledge gap concerning the post-acute brain effects of serotonergic psychedelics. Its findings suggest that changes in brain activity observed just one-day after a high dose psychedelic experience are very different to those found during the acute psychedelic state. Specifically, whereas the acute psychedelic state in healthy volunteers is characterised by modular disintegration^14,15,28^ and global integration^14,19,29^, there are trends towards modular (re)integration and minimal effects on global integration/segregation post psilocybin for depression. Relating the blood flow findings to what has been seen previously in the acute psychedelic state is somewhat more complicated due to inconsistencies in this literature – likely due to analysis approaches and interpretation^14,15,30^: Here we saw decreased CBF bilaterally in the temporal lobes, including the left amygdala one-day post treatment. Decreased *absolute* CBF in subcortical and high-level association cortices have been previously reported with intravenous (I.V.)^15^ and now oral psilocybin^30^ but increased CBF and metabolism have also been reported with I.V. LSD^14^, oral psilocybin^31^, and oral ayahuasca^32^.

Much recent research has focused on the involvement of the default-mode network in psychiatric disorders^33^, and particularly depression^34,35^. We previously observed *decreased* DMN functional integrity under psilocybin^15^ and LSD^14^, and others have with ayahuasca^28^. Here however, *increased* DMN integrity was observed one-day post treatment with psilocybin, both via seed (i.e. vmPFC and sgACC) and network-based approaches. Previous work has suggested that increased DMN integrity may be a marker of depressed mood and specifically, depressive rumination^34,36^. On this basis, increased DMN integrity post psilocybin may be surprising. The post-treatment increases in within-DMN RSFC and sgACC-PCC RSFC did not relate to symptom improvements but vmPFC-ilPC RSFC did (see Fig. 3). This apparent divergence from previous findings^36,37^ is intriguing, and deserves further discussion (below).

It should be noted that findings of elevated within-DMN RSFC in depression are not entirely consistent in the literature^38–41^. For example, using a DMN-focused analysis, precuneus-DMN RSFC^39^ was found to be lower in patients than in healthy controls, and normalised after treatment with electroconvulsive therapy (ECT) - and only in responders^39^ – consistent with the present findings. Lower precuneus-DMN RSFC in depression was also seen in a separate study and the degree of this abnormality correlated with autobiographical memory deficits^40^. In another study, lower PCC-dmPFC and PCC-ilPC RSFC were seen in first-episode depressed patients relative to healthy controls^41^. In the present study, we saw increased within-DMN RSFC post treatment with psilocybin, and increased vmPFC-bilateral ilPC RSFC was predictive of treatment response at 5 weeks (Fig. 3). These findings suggest a commonality in the antidepressant action of ECT and psilocybin^39^ in which DMN integrity is decreased acutely (at least by the latter^14,15,28^) and increased (or normalised) post-acutely, accompanied by improvements in mood. This process might be likened to a ‘reset’ mechanism in which acute modular disintegration (e.g. in the DMN) enables a subsequent re-integration and resumption of normal functioning.

Recent meta-analyses of studies of resting-state CBF in depression have yielded relatively mixed results^34,42^, although findings of increased thalamic^34,42^ and sgACC metabolism are relatively consistent^34^. Here, we did not find any post-treatment changes in thalamic or sgACC CBF with psilocybin, either in whole-brain or ROI-based analyses. We did observe decreased CBF bilaterally in the temporal cortex however, including the left medial temporal lobe and specifically, the left amygdala. Given previous findings of elevated resting-state amygdala CBF and metabolism in mood disorders^25,43,44^, the reduction in amygdala CBF observed here, and its relation to symptom severity, could be viewed as a possible remediation effect. Moreover, generalised decreases in CBF are (again) consistent with what has been previously reported with ECT^45^, i.e. most studies have documented an increase in CBF in the acute ‘ictal’ state, including in the amygdala^45^; however, the post-ictal period is characterised by decreased CBF, and often in those regions that were most perfused during seizure^45^. Acutely increased CBF has previously been reported with ayahuasca^32^ and LSD^15^ and increased glucose metabolism has been observed in the acute state with oral^31^ but not I.V. psilocybin^15^. Thus, a post-acute *reversal* of acute increases in CBF could be seen as consistent with the post-treatment ‘reset’ mechanism proposed above – although recent work has laid into question whether oral psilocybin does indeed cause increases in brain absolute CBF^30^. It would be challenging (but not impossible) to carry out acute *and* post-acute imaging in future trials of psilocybin for depression, and this may be necessary if the ‘reset’ model is to be properly tested. In such a study, we would advise focusing on BOLD RSFC (and perhaps simultaneous EEG-related measures) rather than CBF, due to RSFC and EEG offering more direct and reliable indices of brain activity and function than more difficult to interpret measures such as CBF. The inclusion of a healthy control group, exposed to a consistent treatment procedure, would further strengthen the design of such a study, as would the inclusion of a placebo and/or active comparator arm.

The present study’s other major positive finding was a decrease in RSFC between the bilateral parahippocampus and the PFC, an effect that (like increased vmPFC-ilPC RSFC) was predictive of treatment response at 5 weeks. Curiously, a post-hoc exploratory analysis suggested that acute ‘peak’ or ‘mystical-type’ experiences under psilocybin may mediate the post-acute changes in parahippocampal RSFC (including decreased PH-PCC RSFC). Focusing on parahippocampal-PFC RSFC, this has generally been found to be elevated in depression^46^, and consistently so across the duration of a resting-state scan^47^. Prefrontal-limbic circuitry has been linked with top-down suppression of affective responsiveness^48^ and lower resting-state amygdala-vmPFC RSFC in combination with amygdala hyperfusion was found to relate to state-anxiety in healthy individuals^43^, corroborating separate findings^49^. Seven days of citalopram has been found to reduce amygdala-vmPFC^50^ and dorso-medial PFC-left hippocampal RSFC^51^ in healthy volunteers, somewhat consistent with the present findings.

In conclusion, here we document for the first time, changes in resting-state brain blood flow and functional connectivity post-treatment with psilocybin for treatment-resistant depression. Decreased blood flow was found to correlate (in the amygdala) with reductions in depressive mood. Increased within-DMN RSFC was observed post-treatment, using both seed and network-based analyses, and specific increases in RSFC between the vmPFC and bilateral ilPC nodes of the DMN were greatest in individuals who maintained treatment-response at 5 weeks. Finally, decreased PH-PFC RSFC was observed post-treatment and this was also predictive of treatment-response at 5 weeks. An exploratory post-hoc analysis revealed that acute ‘peak’ or ‘mystical’ experience during the high-dose psilocybin session was predictive of these changes in PH RSFC.

This study is limited by its small sample size and absence of a control condition. Moreover, correction for multiple testing was applied to the full RSN but not the specific (hypothesis-based) ROI analyses. Future research with more rigorous controls should serve to challenge and develop the present study’s findings and inferences. Assessing the relative contributions of, and potential interactions between, the different treatment factors (e.g. the drug and the accompanying psychological support) may be a particularly informative next step.

## Method

This study was approved by the National Research Ethics Service (NRES) committee London – West London and was conducted in accordance with the revised declaration of Helsinki (2000), the International Committee on Harmonisation Good Clinical Practice (GCP) guidelines and National Health Service (NHS) Research Governance Framework. Imperial College London sponsored the research which was conducted under a Home Office license for research with schedule 1 drugs. The Medicines and Healthcare products Regulatory Agency (MHRA) approved the study. All patients gave written informed consent, consistent with GCP.

### Imaging vs clinical outcomes

To explore relationships between significant imaging outcomes and the main clinical outcomes, we chose to focus on changes in depressive symptoms from: 1) pre-Treatment to scan 2 (i.e. one-day post-treatment), and 2) pre-Treatment to 5 weeks post-Treatment. The primary clinical outcome measure, the 16-item Quick Inventory of Depressive Symptoms (QIDS-SR16) was chosen for this purpose. Relationships between imaging outcomes and contemporaneous decreases in depressive symptoms were calculated using a standard Pearson’s r, and relationships with the longer-term (i.e. at 5 weeks post-treatment) changes in depressive symptoms were calculated by splitting the sample into responders (>50% reduction in QIDS-SR16 scores) and non-responders at this time-point, and then performing a one-tailed t-test on the relevant imaging outcomes (one-tailed as directionality was unequivocally implied by the direction of the significant imaging outcome). We used a revised version of the QIDS-SR16 for 24-hour measurement for the post-treatment scan in order to get a contemporaneous, state-related index of depressive symptoms at this time-point.

### Anatomical Scans

Imaging was performed on a 3 T Siemens Tim Trio using a 12-channel head coil at Imanova, London, UK. Anatomical images were acquired using the ADNI-GO (Alzheimer’s Disease Neuroimaging Initiative, Grand Opportunity^52^) recommended MPRAGE parameters (1 mm isotropic voxels, TR = 2300 ms, TE = 2.98 ms, 160 sagittal slices, 256 × 256 in-plane FOV, flip angle = 9 degrees, bandwidth = 240 Hz/pixel, GRAPPA acceleration = 2).

### BOLD fMRI Resting State Acquisition

T2*-weighted echo-planar images (EPI) were acquired for the functional scan using 3 mm isotropic voxels, TR = 2000 ms, TE = 31 ms, 36 axial slices, 192 mm in-plane FOV, flip angle = 80 degree, bandwidth = 2298 Hz/pixel, GRAPPA acceleration = 2, number of volumes = 240, 8 min.

### BOLD Pre-processing

Four different but complementary imaging software packages were used to analyse the fMRI data. Specifically, FMRIB Software Library (FSL)^53^, AFNI^54^, Freesurfer^55^ and Advanced Normalization Tools (ANTS)^56^ were used. Fifteen subjects were used for this analysis: one subject was discarded from the analysis due to an injury in parietal cortex and three subjects were discarded due to high levels of head movement. Principally, motion was measured using frame-wise displacement (FD)^57^. The criterion for exclusion was subjects with >20% scrubbed volumes with a scrubbing threshold of FD = 0.5. For the 15 subjects that were used in the analysis, there was no significant difference in the mean FD (meanFD_before_ = 0.179 ± 0.088, meanFD_after_ = 0.158 ± 0.084, p = 0.23). The mean percentage of scrubbed volumes for before and after treatment was 4.6 ± 5% and 3.5 ± 5.2%, respectively (p = 0.56). The maximum of scrubbed volumes for before and after treatment was 17.3% and 17.7%, respectively. The following pre-processing stages were performed: 1) removal of the first three volumes; 2) de-spiking (3dDespike, AFNI); 3) slice time correction (3dTshift, AFNI); 4) motion correction (3dvolreg, AFNI) by registering each volume to the volume most similar, in the least squares sense, to all others (in-house code); 5) brain extraction (BET, FSL); 6) rigid body registration to anatomical scans (BBR, FSL); 7) non-linear registration to 2 mm MNI brain (Symmetric Normalization (SyN), ANTS); 8) scrubbing^58^ - using an FD threshold of 0.5, scrubbed volumes were replaced with the mean of the surrounding volumes. 9) spatial smoothing (FWHM) of 6 mm (3dBlurInMask, AFNI); 10) band-pass filtering between 0.01 to 0.08 Hz (3dFourier, AFNI); 11) linear and quadratic de-trending (3dDetrend, AFNI); 12) regressing out 9 nuisance regressors (all nuisance regressors were band-pass filtered with the same band-pass filter as above): out of these, 6 were motion-related (3 translations, 3 rotations) and 3 were anatomically-related (not smoothed). Specifically, the anatomical nuisance regressors were: 1) ventricles (Freesurfer, eroded in 2 mm space), 2) draining veins (DV) (FSL’s CSF minus Freesurfer’s Ventricles, eroded in 1 mm space) and 3) local white matter (WM) (FSL’s WM minus Freesurfer’s subcortical grey matter (GM) structures, eroded in 2 mm space). Regarding local WM regression, AFNI’s 3dLocalstat was used to calculate the mean local WM time-series for each voxel, using a 25 mm radius sphere centred on each voxel^59^.

### Seed-based RSFC

Based on prior hypotheses, 4 seeds were chosen for these analyses: 1) the bilateral PH, vmPFC, sgACC and bilateral amygdala. The PH seed was constructed by combining the anterior and posterior parahippocampal gyrus from the Harvard-Oxford probabilistic atlas, which was then thresholded at 50%. The vmPFC seed was the same as one previously used by our team in analyses of the acute effects of LSD^60^, psilocybin^61^ and MDMA^62^. The sgACC seed was a 5 mm sphere centred at ±2 28 -5 (MNI_152 coordinates) based on^63^. Bilateral amygdala seed was based on Harvard-Oxford probabilistic atlas, threshold at 50%. Mean time-series were derived for these seeds for each RS scan. RSFC analyses were performed using FSL’s FEAT for each subject. Pre-whitening (FILM) was applied. A higher level analysis was performed to compare pre-treatment and post-treatment conditions using a mixed-effects GLM (FLAME 1 + 2), cluster corrected (z > 2.3, p < 0.05). MRIcron was used to display the results.

### Resting State Networks (RSN)

RSNs were derived using Independent Component Analysis (ICA) performed on data acquired separately as part of the Human Connectome Project (HCP)^64^. This procedure is identical to one used previously with LSD^60^. Briefly, 20 independent components (ICs) were derived, of which the same 12 functionally meaningful RSNs were identified, namely: medial visual network (VisM), lateral visual network (VisL), occipital pole network (VisO), auditory network (AUD), sensorimotor network (SM), default-mode network (DMN), parietal cortex network (PAR), dorsal attention network (DAN), salience network (SAL), posterior opercular network (POP), left fronto-parietal network (lFP) and right fronto-parietal network (rFP).

### Integrity (within-RSN RSFC)

Network integrity was calculated for each RSN for both pre-treatment and post-treatment. All 20 HCP ICA components were entered into FSL’s dual regression analysis^65^. The first step of the dual regression used the components as regressors applied to the 4D BOLD datasets for each subject, resulting in a matrix of time-series for each ICA. The second step involved regressing these time-series into the same 4D scan data to get a subject-specific set of spatial maps (parameter estimate (PE) images). For each subject and for each condition, within each of the 12 RSNs of interest (threshold = 3), the mean PE across voxels was calculated. This mean PE represents the integrity value. Subsequently, paired t-tests were used to calculate the difference in integrity between conditions for each RSN (Bonferroni corrected for 11 RSNs, with no correction for DMN as we had a prior hypothesis).

### Segregation (between-RSN RSFC)

Between-RSN RSFC was calculated in a similar manner to previous analyses involving acute LSD^60^ and psilocybin^66^. Specifically, a 12 × 12 matrix was constructed representing RSFC between different RSN pairs. For each subject and for each condition, the time-series for the relevant pair of RSNs, was entered into a GLM, resulting in a PE value representing the strength of functional connectivity between them. GLM was used rather than correlation coefficients because differences between Pearson’s correlations could be a result of either signal or noise differences; therefore, it is preferable to perform regression and look for pre-treatment and post-treatment differences on the PE^67^. The GLM was estimated twice: 1) each RSN as a dependant variable in one model, and 2) each RSN as an independent variable in the second model. These two PE values were then averaged together, to generate a symmetric 12 × 12 matrix (Fig. 4b). Three 12 × 12 matrices were created as follows: 1) the group mean PE values for pre-Treatment treatment, 2) the group mean PE values for post-Treatment treatment, and 3) paired t-test to compare the PE values for the two conditions, pre-Treatment and post-Treatment treatment (two-tailed, 5000 permutations).

## Electronic supplementary material

Dataset 1
