# Supplementary material for: Psilocybin for treatment-resistant depression: fMRI-measured brain mechanisms
Source: Sci Rep. 2017 Oct 13;7:13187. doi: 10.1038/s41598-017-13282-7 (PMC5640601; doi:10.1038/s41598-017-13282-7)
Supplement: Supplementary file 1 — Dataset 1 [file 41598_2017_13282_MOESM1_ESM.docx]

**Supplementary file**

**Title: Psilocybin for treatment-resistant depression: fMRI-measured brain mechanisms**

**Robin L Carhart-Harris^a1^, Leor Roseman^a,b^, Mark Bolstridge^a^, Lysia Demetriou^e^, J Nienke Pannekoek^a^, Matthew B Wall^e^, Mark Tanner^e^, Mendel Kaelen^a^, John McGonigle^e^, Kevin Murphy^c^, Robert Leech^b^, H Valerie Curran^d^, David J. Nutt^a^**

^a^ Psychedelic Research Group, Centre for Psychiatry, Department of Medicine, Imperial College London, W12 0NN, London, UK

^b^ Computational, Cognitive and Clinical Neuroscience Laboratory (C3NL), Department of Medicine, Imperial College London, W12 0NN, London, UK

^c^ Cardiff University Brain Research Imaging Centre (CUBRIC), Department of Psychology, CF10 3AT, Cardiff, UK

^d^ Clinical Psychopharmacology Unit, University College London, WC1E 6BT, London, United Kingdom

^e^ Imanova Centre for Imaging Sciences, Burlington Danes Building, Hammersmith Hospital, Du Cane Road, London, W12 0NN, UK

^1^ To whom correspondences should be addressed. Email: r.carhart-harris@imperial.ac.uk

The authors declare no competing financial interests.

**Supplementary data table**


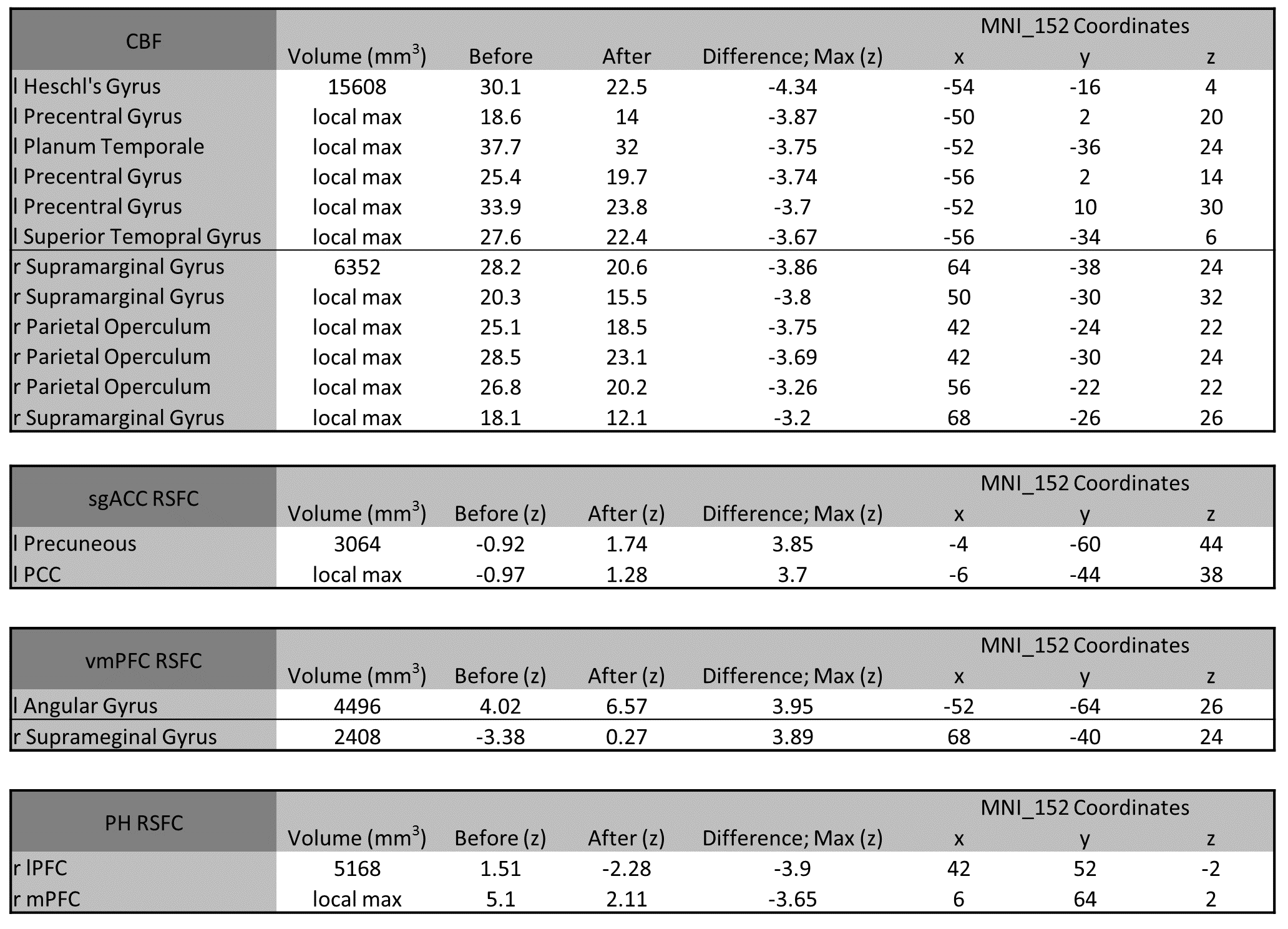


**Table 1.** Regions showing decreased CBF, increased sgACC RSFC, increased vmPFC RSFC and decreased PH RSFC post treatment. The before (pre) and after (post) treatment columns report the z value of each condition separately in the same point as Max.

**Supplementary analysis**

Inspired by a reviewers’ request, we looked for correlation between the quality of the acute experience – with a specific focus on the so-called ‘mystical experience’ and changes in RSFC in a whole brain voxel based analysis. The index of mystical experience we used here was the Oceanic Boundlessness (OB) dimension of the 5D-ASC (Studerus et al., 2010) and the parahippocampus was chosen as the primary region of interest for the RSFC analysis - due to previous work implicating its circuitry in phenomena relevant to the ‘mystical experience’ (Carhart-Harris et al., 2016). We found a negative correlation between OB and the changes in RSFC of parahippocampus with a number of regions (precuneous, frontal pole, insular cortex, putamen and amygdala) (cluster correction, z > 2.3, p < 0.05). The nature of this relationship was such that patients who scored high on OB had the greatest decreases in RSFC. Since PH-PFC RSFC was decreased as a main result (fig of main paper), this finding makes some intuitive sense. We recommend caution in making inferences on this result however; the analysis was conceived post-hoc nature and that the patients who scored lowest on OB actually had an increase in PH RSFC in the relevant regions – which is inconsistent with the main result reported in the manuscript, i.e. decreased PH-PFC RSFC.


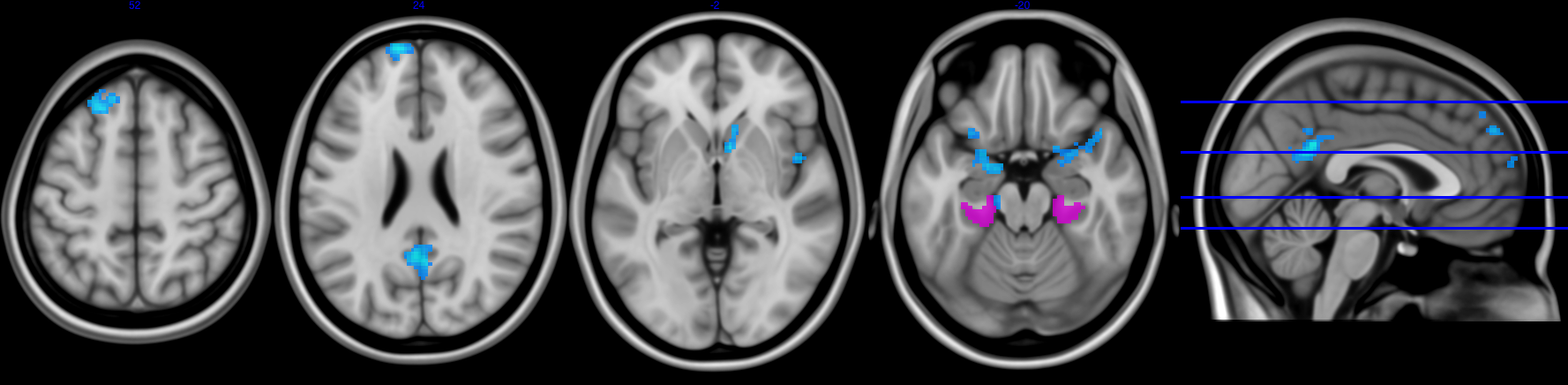

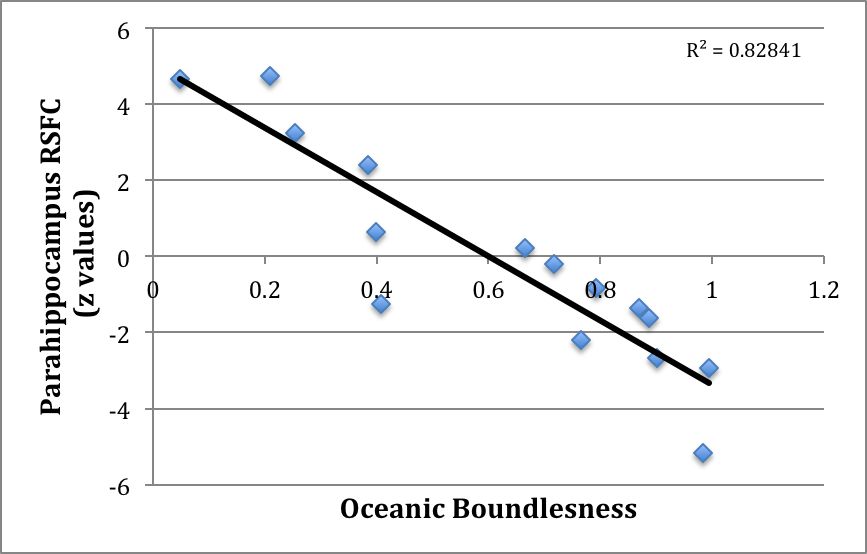


**Figure S1.** Statistical maps show regions where post-treatment changes in parahippocampal RSFC correlate with ratings of ‘oceanic boundlessness’ (OB) (z = 2.3, p < 0.05). The correlation plot shows the distribution of values. It can be seen that some patients actually had increased PH-RSFC in the relevant regions – and that these had the lowest OB scores.

**References**

Carhart-Harris RL, Muthukumaraswamy S, Roseman L, et al. (2016) Neural correlates of the LSD experience revealed by multimodal neuroimaging. *Proc Natl Acad Sci U S A* 113: 4853-4858.

Studerus E, Gamma A and Vollenweider FX. (2010) Psychometric evaluation of the altered states of consciousness rating scale (OAV). *PLoS One* 5: e12412.
